# Feasibility of Very Early Identification of Cardiogenic Shock by Semi-automated Ultrasound Exam in the Emergency Department

**DOI:** 10.7759/cureus.30927

**Published:** 2022-10-31

**Authors:** Gabriel Morales, Adeyinka Adedipe, Sophie Morse, James McCabe, Claudius Mahr, Graham Nichol

**Affiliations:** 1 Department of Emergency Medicine, University of Washington, Seattle, USA; 2 Department of Medicine, Division of Cardiology, University of Washington, Seattle, USA

**Keywords:** echocardiogram, resuscitation, shock, lung ultrasound (lus), velocity time integral, point-of-care-ultrasound, cardiogenic shock

## Abstract

Background

Cardiogenic shock (CS) is critical end-organ hypoperfusion due to reduced cardiac output. Early therapy, such as vasoactive agents or the initiation of mechanical circulatory support (MCS), requires early diagnosis and is associated with better outcomes. A novel ultrasound platform (GE Healthcare, Milwaukee, WI) has semi-automated imaging software (SAIS), which could simplify the point-of-care ultrasound (POCUS) diagnosis of CS. We assessed the feasibility of using POCUS with SAIS in patients in shock, determined the ability of SAIS to identify the subset of patients with CS, and described the process and outcome of care of patients with vs. without CS after presenting to Emergency Department (ED) with hypotension.

Methods

This prospective case-control study was conducted at an urban ED. Physicians with prior POCUS education received one hour of training with the study device. The qualitative ejection fraction was determined by visual assessment. SAIS measurements of hemodynamics were made with the study device and included left ventricle outflow tract velocity time integral (LVOT VTI), inferior vena cava collapsibility or distensibility indices, and pulmonary B-line assessment. ED patients with a systolic blood pressure ≤ 90 mmHg or need for a vasopressor initiation in the ED were enrolled. The diagnosis of CS was determined by a medical record review. All data were summarized descriptively.

Results

Twenty-nine cases underwent POCUS, and 87 controls did not. Baseline characteristics, process, and outcome of care were similar between groups. Seventy-nine percent (79%) of cases had a complete POCUS with SAIS. Of these, 55% had reduced LVOT VTI, 38% had IVC collapsibility <50%, and 48% of cases had a B-line pattern consistent with pulmonary edema. The mean LVOT VTI for cases with CS was 9.4± 5.4 cm; the mean LVOT VTI for cases without CS was 15.2 ± 6.0 cm. Among patients who did not undergo POCUS, 31 (36%) had a formal echocardiogram, and eight (9%) had a final diagnosis of cardiogenic shock during hospitalization.

Conclusion

Physicians with one hour of platform-specific training were able to implement POCUS with SAIS among patients who present with shock. POCUS with SAIS may aid in the early recognition of CS.

## Introduction

Cardiogenic shock (CS) is defined as a state of critical end-organ hypoperfusion due to reduced cardiac output [1-7]. Timely identification of CS in undifferentiated emergency department (ED) patients can be challenging, however, point-of-care echocardiography enables the immediate assessment of cardiac function and helps with early identification of CS [8-10]. Point-of-care ultrasound (POCUS) assessment can be performed by emergency physicians [11-13], can aid in the diagnosis of CS, and may influence treatment [14,15]. Ultrasound measurements of pulmonary B-lines enable the assessment of pulmonary congestion while measurements of inferior vena cava (IVC) collapsibility and distensibility evaluate volume status [16-18]. POCUS assessment of left ventricular (LV) ejection fraction can be accurately performed in the ED; however, there remains some variability across emergency providers in the ability to perform and interpret bedside ultrasound [19-21]. Thus, early identification of ED patients with CS at the time of presentation requires a broad hemodynamic assessment of multiple organ systems and can be challenging and operator-dependent. A simple, reliable method of identifying those patients with CS among ED patients in undifferentiated shock could decrease the time to recognition of CS with the potential to improve outcomes.

In the present study, the primary objective was to determine the feasibility of ED physicians using POCUS with a semi-automated imaging software protocol (SAIS) to screen patients with undifferentiated hypotension for underlying cardiogenic shock. The Venue ultrasound platform (GE Healthcare Inc., Milwaukee, WI) provides semi-automated imaging and interpretation software that could assist emergency providers in identifying patients in CS. We hypothesized that ED physicians could perform POCUS with SAIS on patients in shock to capture advanced hemodynamic measurements, potentially identifying a subset of patients with CS. The secondary objective was to describe the process and outcome of care in enrolled patients compared to selected controls.

## Materials and methods

Design and setting

This prospective case-control study was conducted at an urban tertiary care hospital. Patient-case enrollment took place from October 2018 to March 2019 while the controls were enrolled from July 2018 to August 2019. We planned to enroll 30 patients with undifferentiated hypotension as recommended for pilot studies [22] and retrospectively enrolled 90 controls with a 3:1 ratio. Cases were evaluated using the POCUS imaging protocol defined a priori; controls were not evaluated with the POCUS imaging protocol.

Population

Included cases were a convenience sample of patients who had at least two systolic blood pressure measurements ≤ 90 mmHg (noted at triage, in nursing notes, or on bedside monitors) or who required any vasopressor medications at any point during their ED stay. Patients were identified by on-duty study physicians and enrolled consecutively. Eligible controls were patients who presented to the ED during the study period, had at least two systolic blood pressure measurements ≤ 90 mmHg (noted at triage, in nursing notes, or on bedside monitors), or who required any vasopressor medications at any point during their ED stay, did not undergo POCUS with SAIS and were identified by a review of administrative records. Excluded from both groups were patients with an obvious traumatic injury. Controls were retrospectively matched to cases based on sex and age at the time of the ED visit with a 3:1 ratio.

Physician education of study procedures

Physicians with >3 years of experience and who had previously completed the standard American emergency medicine (EM) residency-based ultrasound curriculum during residency were eligible to participate. The study physician team included four senior EM residents and six faculty. Physicians completed a one-hour hands-on session with the study device, including general machine functionality and familiarization with semi-automated functions. The training was performed by an ultrasound fellowship-trained EM faculty member.

Imaging protocol and semi-automated functions

**Figure 1:**
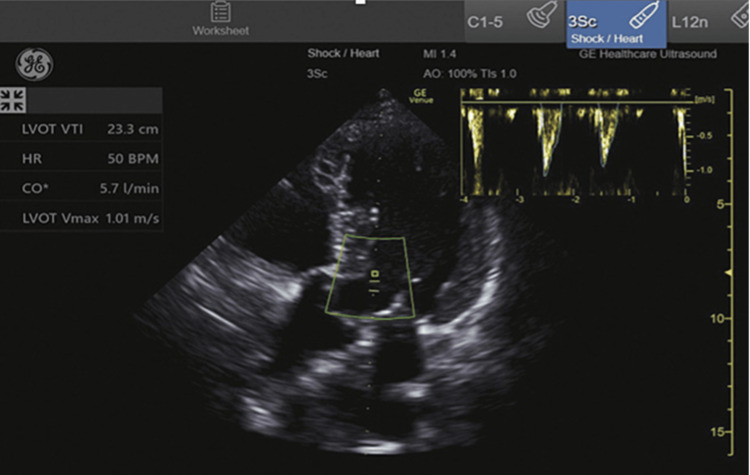
Point of care ultrasound with semi-automated software platform with LVOT VTI LVOT VTI: left ventricle outflow tract velocity time integral

**Figure 2:**
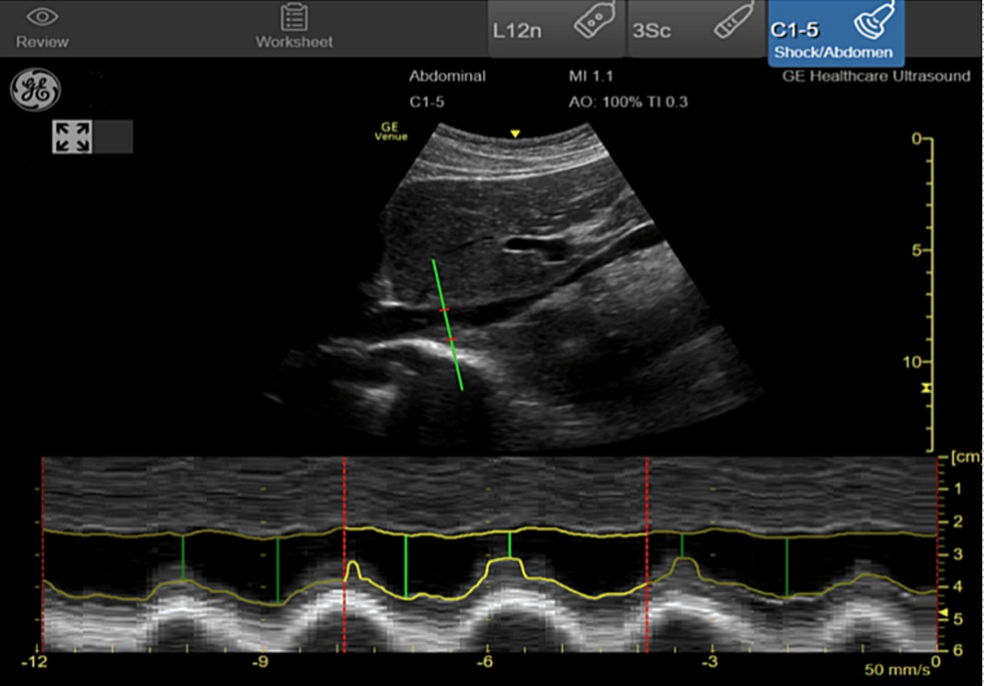
Point of care ultrasound with semi-automated software platform with IVC IVC: inferior vena cava

**Figure 3:**
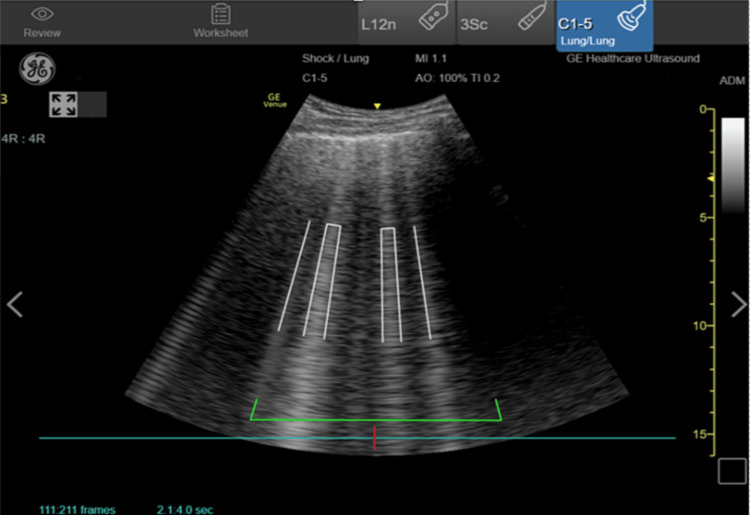
Point of care ultrasound with semi-automated software platform with B-lines

Measurements included LV ejection fraction, LV outflow tract velocity time integral (LVOT VTI), IVC collapsibility or distensibility indices, and the number of pulmonary B-lines. The LV ejection fraction was visually estimated as normal (>50%), reduced (30 to 50%), or severely reduced (< 30%). The measurements of LVOT VTI, IVC collapsibility or distensibility, and B-line quantification were semi-automated imaging measurements obtained with the GE Venue (General Electric Healthcare Inc., Milwaukee, WI), an FDA-approved ultrasound system designed to make advanced cardiac measurements using artificial intelligence and machine learning. VTI is an estimation of the distance that a column of blood travels in one systolic beat and is used to calculate stroke volume and cardiac output. The LVOT VTI measurement on the GE Venue is obtained while imaging the heart in an apical 5-chamber view (Figure 1). When the user activates the auto VTI button, a pulse wave Doppler sampling gate is automatically placed over the aortic annulus of the LV (Figure 1). If the user disagrees with the location of the sampling gate, they can manually reposition it to a preferred location. After three cardiac cycles, the Venue system automatically traces three pulse wave Doppler waveforms and averages them to obtain LVOT VTI in real-time. The apical five-chamber view is a variation of the apical four-chamber view (Video 1). The LVOT and aortic valve are brought into view by rotating the probe clockwise and angling slightly anteriorly. If the user has previously measured the LVOT diameter in a parasternal long-axis view (Video 2), Venue calculates stroke volume and cardiac output and displays these measurements on the screen. The auto IVC tool measures collapsibility or distensibility indices from a longitudinal view of the IVC obtained at the cavo-atrial junction (Figure 2). By activating the Auto IVC button, the Venue system determines the edges of the IVC and automatically calculates the change in IVC diameter size during respiratory cycles using the maximum and minimum diameters identified (Figure 2). The collapsibility or distensibility index is displayed on the screen in real-time and updated breath-to-breath. When the Auto B-Line tool is activated, pulmonary B-lines are highlighted on the screen (Figure 3). Venue will then count the number of B-lines displayed and provide an overall B-line score when the user scans through four different lung zones on each hemithorax in a systematic fashion. Prior to FDA approval, the Venue system’s semi-automated features were internally and externally validated on human subjects comparing physicians with certified sonographers. For the present study, an ultrasound fellowship-trained faculty member reviewed all images to ensure the appropriateness of SAIS measurements. Information obtained from POCUS SAIS was not used to make clinical decisions during the study.

**Video 1:** Apical four-chamber view

**Video 2:** Parasternal long-axis view

Data and definitions

Low cardiac output was defined as a visual estimation of ejection fraction as reduced or severely reduced or an LVOT VTI < 18 cm. Volume non-responders were defined as IVC collapsibility index of < 50% in spontaneously breathing patients or a distensibility index of < 18% in mechanically ventilated patients. Pulmonary congestion was defined as the presence of > 3 B-lines present in both hemithoraces.

Two study team members blinded to POCUS results performed chart abstraction. Baseline and covariate factors were abstracted from the electronic medical record, including age, gender, history of heart failure, chest pain, dyspnea, fever (temperature > 38.3 C), electrocardiographic ischemia (ST-segment depression > 1 mm, ST-segment elevation > 2 mm or T-wave flattening or inversion), elevated troponin (> 0.03 ng/mL), and use of a vasopressor. ECG data were abstracted retrospectively and assessed by a study investigator, including rate, rhythm, conduction, ischemia, presence of Q waves, and serial changes. In-hospital outcomes for both cases and controls were assessed including mortality, vasopressors > 6 hours, inpatient echo, cardiac catheterization, CS, and mechanical circulatory support (MCS).

There is no clear gold standard definition for CS. In this study, we screened for CS in patients who presented to the ED with a systolic blood pressure of ≤ 90 mmHg for any period of one hour within 48 hours of enrollment or who required one hour of vasopressors as documented in the patient’s electronic medical record. A study investigator retrospectively determined each patient’s underlying cause of shock, for both cases and controls, by using available information in the patient’s medical record at discharge (e.g., echo reports, diagnostic testing results, physician notes), to identify CS versus non-CS etiologies with adjudication performed by the study team lead. Non-CS etiologies of shock included distributive, hypovolemic, or obstructive shock.

Outcome measures and analysis

The primary objective was measured as the percentage of cases that had a complete imaging protocol performed. All data were summarized descriptively. The information available from SAIS was not used to make clinical decisions.

Ethics

The University of Washington Institutional Review Board approved this study with a waiver of documented written consent under minimal risk criteria, approval number UW IRB STUDY00004110.

## Results

A total of 117 patients were enrolled (30 cases and 87 controls). One case was excluded due to the retrospective diagnosis of traumatic neurogenic shock, leaving 29 cases of POCUS with SAIS and 87 controls (Table 1). Among cases, 62% had male gender; were aged 58 ± 17.2 years; with a BMI of 24.1±5.4. Among controls, 62% had male gender; were aged 58 ± 17.0 years; with a BMI of 27.6±9.9. Overall, 12 (10%) patients had a final diagnosis of CS (four cases and eight controls). Eighty-three percent (83%) of patients with CS had a troponin level higher than 0.1 ng/mL (normal <0.03 ng/mL) versus 11% of those without CS (Table 2). Among patients with CS, 41% had a history of heart failure, 50% had ischemic ECG patterns, 42% had pathologic Q waves, and 50% had evidence of T wave abnormality. Fever was absent among all CS patients.

**Table 1:** Patient demographics for cases and controls ACE: angiotensin-converting enzyme

	Cases	Controls	Overall
N	29	87	116
Age (mean & SD)	58±17.2	58±17.0	58±17.2
Male, n (%)	18 (62)	54 (62)	72 (62)
BMI (mean & SD)	24.1±5.4	27.6±9.9	26.4±8.8
Intubated, n (%)	10 (34)	15 (17)	25 (22)
Pre-existing Conditions			
History of Heart Failure, n (%)	7 (24)	19 (22)	26 (22)
Renal Insufficiency, n (%)	12 (41)	36 (41)	48 (41)
Hepatic Insufficiency, n (%)	2 (7)	6 (7)	8 (7)
Medications Prior to ED			
Beta Blockers, n (%)	12 (48)	24 (28)	36 (31)
ACE Inhibitors, n (%)	1 (4)	9 (10)	10 (9)
Shock Final Diagnosis, n (%)			
Cardiogenic	4 (14)	8 (9)	12 (10)
Hypovolemic	8 (27)	26 (30)	34 (29)
Distributive	17 (59)	51 (59)	68 (59)
Obstructive	0 (0)	2 (2)	2 (2)

**Table 2:** Patient characteristics at presentation BNP: brain natriuretic peptide; CS: cardiogenic shock

	Cases			Controls			Overall
	With CS	Without CS	All	With CS	Without CS	All	
Total (n)	4	25	29	8	79	87	116
Dyspnea, n (%)	0 (0)	5 (20)	5 (17)	4 (50)	29 (37)	33 (38)	38 (33)
Troponin >0.1 ng/mL, n (%)	2 (50)	2 (8)	4 (14)	8 (100)	9 (11)	17 (20)	21 (18)
BNP During ED Visit (ng/L mean & SD)	1557±3387	931±1126	1432±3022	1258±2224	771±519	982±1964	1092±2233
ECG Ischemia, n (%)	2 (50)	5 (20)	7 (24)	4 (50)	21 (27)	25 (29)	32 (28)
Measured Fever, n (%)	0 (0)	4 (16)	4 (14)	0 (0)	5 (6)	5 (6)	9 (8)
Heart Rate (mean)	74±35	88±32	86 ±32	128±39	97±26	99±29	95±31
Pathologic Q Waves Present, n (%)	2 (50)	3 (12)	5 (17)	3 (38)	4 (10)	8 (9)	13 (11)
T Wave ischemia, n (%)	2 (50)	4 (16)	6 (20)	4 (50)	16 (20)	21 (24)	27 (23)
T Wave Location	NA	Inferior	Inferior	NA	Lateral	Lateral	Lateral
ECG Ventricular Hypertrophy, n (%)	0 (0)	0 (0)	0 (0)	2 (25)	1 (1)	3 (3)	3 (3)
Second ECG With Ischemic Change, n (%)	1 (25)	2 (8)	3 (10)	2 (25)	4 (5)	6 (7)	9 (8)
Paced, n (%)	0 (0)	1 (13)	1 (3)	1 (13)	0 (0)	1 (1)	2 (2)
Rhythm (mode)	NA	Normal Sinus	Normal Sinus	Sinus Tachycardia	Normal Sinus	Normal Sinus	Normal Sinus

Among cases, 28/29 (97%) had a visually estimated ejection fraction obtained, three (10%) of which were reduced or severely reduced (Table 3). Individually, LVOT VTI measurement, IVC indices, and pulmonary B-lines quantification were obtained in 83%, 97%, and 100% of cases, respectively. In total, 23 cases (79%) had a complete POCUS imaging protocol performed, of which 55% had reduced LVOT VTI, 38% had IVC collapsibility <50%, and 48% had a B-line pattern consistent with pulmonary edema. Cases identified with CS as a final diagnosis had a mean LVOT VTI of 9.4±5.4 cm versus 15.2±6.0 cm in cases without CS. All cases with CS were intubated and one (25%) had IVC distensibility <18%. Three or more B lines in bilateral hemithoraces were identified in 50% of CS cases and 40% of non-CS cases.

**Table 3:** Ultrasound feasibility data LVOT VTI: left ventricle outflow tract velocity time integral; IVC: inferior vena cava; CS: cardiogenic shock

Cases	Final Diagnosis CS*, n=4	Final Diagnosis No CS, n=25	All, n=29
LVOT VTI Acquired, n (%)	2 (50)	22 (88)	24 (83)
Ejection Fraction, n (%)	4 (100)	24 (96)	28 (97)
IVC Assessment, n (%)	4 (100)	24 (96)	28 (97)
B Lines, n (%)	4 (100)	25 (100)	29 (100)
LVOT VTI in cm, mean+SD	9.4±5.4	15.2±6.0	14.7±6.1
LVOT VTI < 18cm, n (%)	2 (100)	11 (44)	16 (55)
Cardiac Output (L/min, mean+SD)	1.7±0.1	3.9±2.6	3.7±2.6
Stroke Volume (mL, mean+SD)	27±14.1	45.2±25.4	43.7±24.9
3 or More B Lines Detected in >1 Lung Field	2 (50)	10 (40)	14 (48)
B Line Number (mean+SD)	3.3±2.9	1.8±1.8	1.9±1.9
IVC Collapse <50%	0 (0)	12 (48)	11 (38)
IVC Distensibility <18%, n (%)	1 (25)	3 (12)	4 (14)
Vasopressor Use in ED			
Any, n (%)	4 (100)	8 (32)	12 (41)
>1 hour, n (%)	4 (100)	8 (32)	12 (41)
>6 hours, n (%)	4 (100)	7 (28)	11 (38)

Among controls, 31 (36%) underwent formal echocardiogram during their hospitalization. Of these, 42% had LVOT VTI measured, 19% had a cardiac output measured, and 68% had a stroke volume measured. Eight controls (9%) had a final diagnosis of cardiogenic shock during hospitalization.

Overall, three patients underwent cardiac catheterization during hospitalization, and none received a mechanical circulatory support device during their visit (Table 4). The average length of stay for patients with CS was 7.0±5.4 days compared to 7.1±11.3 days in those without CS. Overall mortality was 10%, whereas the mortality rate of all patients identified as having CS was 42%.

**Table 4:** Outcomes of cases and controls CS: cardiogenic shock

	Cases			Controls				Overall
	With CS, n=4	Without CS, n=25	All, N=29	With CS, n=8	Without CS, N=79	All, n=87	n=116	
Length of Stay, in days mean+SD	9.3±6.7	8.9±14.8	8.9±13.9	5.9±4.8	6.6±10.0	6.5±9.7	7.1±10.8	
Survived to Discharge n (%)	1(25)	24 (96)	4 (13.8)	4 (50)	75 (95)	79 (91)	104 (90)	
Vasopressors > 6 hours, n (%)	4 (100)	7 (28)	11 (38)	8 (100)	21 (27)	29 (33)	40 (34)	
Formal Echo, n (%)	4 (100)	4 (16)	8 (28)	6 (75)	23 (29)	29 (33)	37 (32)	
Cardiac Cath, n (%)	2 (50)	0 (0)	2 (7)	0 (0)	1 (1)	1 (1)	3 (2.6)	

## Discussion

We found that physicians with a brief training period were able to obtain advanced cardiac function measurements using POCUS with SAIS in patients who presented to the emergency department (ED) with shock. The qualitative assessment of EF was obtained in addition to quantitative measurements of LVOT VTI, volume responsiveness, and pulmonary B-line assessment in a large proportion of patients. This suggests that POCUS with SAIS is feasible in an ED setting and may help inform the physician’s clinical impression with precise hemodynamic information. In addition, we found that among patients who presented with hypotension, CS was associated with a lack of fever, ECG abnormalities, and a high mortality rate compared to non-CS patients.

There were several important observations from our study. First, after only one hour of additional training provided by an ultrasound fellowship-trained EM faculty member, complete studies of advanced hemodynamics were obtained in 79% of the cases attempted. This is less time needed and a greater proportion of patients captured than in prior studies [12,13]. We believe that emergency physicians are more likely to perform advanced hemodynamic measurements if the training required is brief and the feasibility of implementation is high. Prior studies have established that using POCUS in ED patients with hypotension decreases diagnostic uncertainty, decreases time to disposition, and reduces time to critical interventions [23-29]. Obtaining more advanced measurements, such as LVOT VTI, requires more skill but provides significant information on the etiology of hypotension and volume responsiveness [30-33]. Improved machine learning and automation of hemodynamic measurements have the potential to reduce error, increase reproducibility, and simplify ongoing assessment.

Second, 75% of patients with CS had a distensibility index of > 18%, suggesting that some patients in CS may be volume responsive. Measuring IVC distensibility can be difficult to perform because it is error-prone to both visually estimate and manually calculate, given the small change that determines fluid responsiveness. In intensive care populations, IVC distensibility > 18% is associated with fluid responsiveness [18,34]. Simplified or automated methods of measuring IVC distensibility may assist in the volume assessment of intubated ED patients. There are certain scenarios in which additional intravenous fluid may be warranted such as right heart failure, right coronary artery ST-elevation myocardial infarction (STEMI), among others, however, the majority of CS is negatively fluid responsive. Differentiating between right and left heart failure, which we did not attempt to do in the present study, could help in further defining the subset of fluid-responsive patients.

Clinical outcomes for patients who presented with hypotension and underwent POCUS with SAIS in the current study warrant further consideration. Four cases enrolled were retrospectively identified as having out-of- hospital-cardiac-arrest (OHCA), two with CS. Some patients experience post-arrest myocardial stunning after resuscitation from OHCA. Usually, this myocardial dysfunction resolves over three days [1-5]. Many centers routinely perform early coronary angiography in patients resuscitated from OHCA, at which time a left ventriculogram can be performed to assess cardiac function. Therefore, it is unclear whether early use of POCUS with semi-automated software would impact outcomes in such cases.

A high proportion of patients with CS had a history of heart failure, elevated troponin > 0.1 ng/ml, ECG ischemia, and absence of fever. In addition, patients in CS were intubated and frequently received vasopressors in the ED. Our findings are similar to a previous study of ED patients with undifferentiated hypotension, which found that independent risk factors for CS included a history of heart failure, presence of shortness of breath, troponin > 0.1 ng/ml, electrocardiogram ischemia, and absence of fever [35]. ECG data demonstrated a high prevalence of pathologic Q waves among patients identified with CS. Patients with CS also had a high prevalence of ischemic t-wave changes. Prior studies of patients with STEMI suggest that the presence of Q waves is independently associated with adverse clinical events including CS [36-39]. Patients who present with Q waves have greater rates of failure of myocardial salvage despite tissue-level reperfusion [36].

Early treatment of CS is predicated on its early identification. Results from the INOVA SHOCK Registry and the National Cardiogenic Shock Initiative (NCSI) demonstrated that increased awareness, rapid identification, early multidisciplinary team activation, rapid initiation of mechanical circulatory support, and hemodynamic-guided management improved outcomes in patients with CS [38,39]. Early interventions that temporize CS may include inotropes and vasodilators to relieve symptoms by reducing cardiac work and increasing myocardial contractility. More effective treatments for CS may include temporary MCS or extracorporeal membrane oxygenation (ECMO). Potential benefits of implementing MCS include restoration of cardiac output in a shock state, unloading the LV, which may limit infarct size and the negative effects of reflow, and an enhanced rate of procedural success of PCI [36]. Importantly, evidence-based practice guidelines recommend the use of MCS or ECMO in patients with CS [37]. Thus, it seems plausible that screening all patients in the ED who present with undifferentiated hypotension for advanced heart failure may decrease the time to MCS implantation and thereby improve overall patient outcomes. This hypothesis requires further study.

Limitations

A key limitation of this work is that there is no consensus gold standard on cutoffs for normal versus abnormal LVOT VTI. In this study, we used 18 cm as a cutoff of high versus low VTI. We observed that the mean LVOT VTI among all cases was 14.7 cm and that 44% of non-CS cases met the criteria for low LVOT VTI. Our findings suggest that a lower VTI cutoff may be necessary to distinguish CS from non-CS in a population of ED patients with hypotension. More research is necessary to establish a reliable range for an LVOT VTI of the ED population with undifferentiated hypotension.

Moreover, another key limitation is that this study enrolled a small number of subjects at a single center. The reproducibility of semi-automated measurements was not evaluated, and SAIS measurements were not compared to echocardiographers for accuracy. All physicians in the present study had a minimum of three years of POCUS training experience, which may not be representative of all emergency physicians. The time required to complete the imaging protocol was not measured. Valvular heart disease, such as aortic stenosis was not evaluated in this study, which may affect POCUS measurements. We did not evaluate whether early identification of CS by POCUS influenced treatment decisions or patient outcomes. We did not evaluate how POCUS was implemented in the control group.

## Conclusions

Early identification of CS in ED patients, through the immediate assessment of cardiac function using point-of-care echocardiography, can be performed by emergency physicians and assist in identifying pulmonary B-lines to determine pulmonary congestion and measure the collapsibility and distensibility of the inferior vena cava to evaluate volume status. Therefore, emergency physicians with minimal platform-specific training can use POCUS with semi-automated analysis to obtain advanced cardiac measurements to identify hypotensive ED patients at risk for CS. Additionally, emergency physicians are more likely to perform POCUS when their training is brief and the implementation is feasible. Further study is necessary to determine if early identification of CS by POCUS with SAIS improves patient outcomes.
